# Kinetics of Local and Systemic Leucocyte and Cytokine Reaction of Calves to Intrabronchial Infection with *Chlamydia psittaci*

**DOI:** 10.1371/journal.pone.0135161

**Published:** 2015-08-07

**Authors:** Annette Prohl, Katharina Wolf, Corinna Weber, Kerstin E. Müller, Christian Menge, Konrad Sachse, Jürgen Rödel, Petra Reinhold, Angela Berndt

**Affiliations:** 1 Institute of Molecular Pathogenesis at ‘Friedrich-Loeffler-Institut’ (Federal Research Institute for Animal Health), Jena, Germany; 2 Institute of Medical Microbiology, Friedrich Schiller University of Jena, Jena, Germany; 3 Ruminant and Swine Clinic at Freie Universität Berlin, Berlin, Germany; University of California, San Francisco, University of California, Berkeley, and the Children's Hospital Oakland Research Institute, UNITED STATES

## Abstract

Infection of cattle with chlamydiae is ubiquitous and, even in the absence of clinical sequeleae, has a quantifiable negative impact on livestock productivity. Despite recent progress, our knowledge about immune response mechanisms capable of counteracting the infection and preventing its detrimental effects is still limited. A well-established model of bovine acute respiratory *Chlamydia* (*C*.) *psittaci* infection was used here to characterize the kinetics of the local and systemic immune reactions in calves. In the course of two weeks following inoculation, leukocyte surface marker expression was monitored by flow cytometry in blood and bronchoalveolar lavage fluid (BALF). Immune-related protein and receptor transcription were determined by quantitative real-time reverse transcription PCR in blood, BALF and lung tissue. An early increase of *IL2RA*, *IL10* and *HSPA1A* mRNA expressions was followed by a rise of lymphocytes, monocytes, and granulocytes exhibiting activated phenotypes in blood. Monocytes showed elevated expression rates of CD11b, CD14 and MHC class II. The rates of CD62L expression on CD8^hi^ T cells in blood and on CD4^+^ T cells in BALF were also augmented and peaked between 2 and 4 dpi. Notably, CD25 antigen expression was significantly elevated, not only on CD8d^im^/CD62L^+^ and CD8^-^/CD62L^+^ cells in blood, but also on granulocytes in blood and BALF between 2–3 dpi. From 4 dpi onwards, changes declined and the calves recovered from the infection until 10 dpi. The findings highlight the effectiveness of rapid local and systemic immune reaction and indicate activated T cells, monocytes and granulocytes being essential for rapid eradication of the *C*. *psittaci* infection.

## Introduction

Members of the family *Chlamydiaceae*, i.e. gram-negative obligate intracellular bacteria, are capable of infecting various mammalian hosts, including humans, and birds. They are often present in the genital, respiratory and intestinal tracts and the conjunctivae, but can also affect other organ systems. Infections with chlamydiae do not always cause clinical disease and often remain clinically inconspicuous. Due to their dependence on host cells for replication, chlamydiae are not detected by routine microbiological diagnostics resulting in a significant underestimation of the actual prevalence.

Seroprevalences of up to 100% in herds tested in different regions worldwide led to the assumption that chlamydial infections are ubiquitous in cattle [1]. *Chlamydia (C*.*) pecorum*, *C*. *abortus*, and *C*. *psittaci* are the species most commonly found in bovines [2–6]. In the last decade, it became obvious that the presence of *Chlamydia* spp. in cattle herds is associated with reduced performance and herd health, even though overt clinical symptoms are mostly absent (reviewed by [7]). *Chlamydia*-infected farms had lower annual milk production per cow [5]. Moreover, chlamydial infections were associated with clinical and subclinical mastitis [8], abortion, premature calving, elevated perinatal calf loss [5], reduced body weight [9], and subclinical pulmonary dysfunction [10] in individual animals. More overt manifestations, e.g. acute respiratory and systemic disease [3,11], as well as keratoconjunctivitis [12], were also reported.

Experimental challenge of calves with *C*. *psittaci* was shown to induce respiratory disease [13]. In a recently established bovine animal model, intrabronchial inoculation with *C*. *psittaci* consistently induced pulmonary lesions and dysfunctions in a dose-dependent manner. For instance, application of 10^8^ inclusion-forming units (ifu) reproducibly set an infection that resulted in acute respiratory disease with fever within 36 hours (h). Clinical signs peaked 2–3 days after inoculation (dpi) and were paralleled by an acute-phase reaction exemplified by a marked increase of lipopolysaccaride-binding protein (LBP) in peripheral blood.

Furthermore, changes in blood cell counts were characterized by an initial increase of total leukocyte numbers, followed by a phase of leukopenia. This was mainly driven by an increase in neutrophilic granulocytes with a regenerative left shift. Changes in blood mirrored increased numbers and percentages of neutrophilic granulocytes in the bronchoalveolar lavage fluid (BALF). Concentrations of eicosanoids and total protein were elevated in the BALF of infected animals. Lung lesions were characterized as fibrinopurulent bronchopneumonia with multifocal areas of necrosis and pleuritis. First signs of regeneration were visible 7 dpi. Animals clinically recovered until 10 dpi and blood and BALF cell counts and LBP levels returned to pre-inoculation values [14–17]. Nevertheless there is an obvious deficit in understanding the underlying mechanisms governing host-pathogen interactions.

Given the comprehensive knowledge gained from experimental *C*. *pittaci* infection of calves, the model was chosen to further scrutinize the local and systemic immune responses, as we hypothesized that their respective factors might be responsible for the relatively quick recovery of the animals from acute respiratory disease. For that reason, the cellular composition of peripheral blood and BALF was examined in more detail with special emphasis on the activation state of leukocyte subsets of the innate and adaptive arms of the immune response. Additionally, transcription of selected mediators and receptors was determined in blood, BALF and lung tissue at different time points after intrabronchial inoculation.

## Animals, Materials and Methods

### Legal conformity and ethics statement

This study was carried out in strict accordance with the German Animal Welfare Act. The protocol was approved by the Committee on the Ethics of Animal Experiments and the Protection of Animals of the State of Thuringia, Germany (“Thüringer Landesamt für Verbraucherschutz”, Bad Langensalza, Germany; Permit Numbers: 04-002/07 and 04-004/11). All experiments were done in a containment at biosafety level 2 under supervision of the authorized institutional Agent for Animal Protection. Bronchoscopy was strictly performed under general anesthesia in infected animals and under light sedation in non-infected controls. During the entire study, every effort was made to minimize suffering.

### Animals

In this prospective and controlled study, 57 conventionally raised calves (Holstein-Friesian, male) were included. Animals originated from one farm without any history of *Chlamydia*-associated health problems. Calves were purchased at the age of 12 to 30 days weighing between 46.2 and 77.6 kg from a herd with no history of chlamydiosis (the herd of origin was regularly checked for the presence of *Chlamydiaceae* spp. by the OIE and National Reference Laboratory for Chlamydiosis over the past eight years). After a quarantine period of at least 21 days and confirmation of a clinically healthy status, animals were included in the study.

To exclude any pre-existing chlamydial infection, each incoming calf was subjected to diagnostic testing by serology and PCR for *Chlamydiaceae* spp. (nasal, ocular, and fecal swabs) immediately after entrance in the premises. A second round of repeated testing was conducted about 3 weeks later, i.e. immediately before challenge. Exclusion of other potential co-infections was performed as described previously [17,18].

Throughout the entire study, animals were reared under standardized conditions (room climate: 18-20°C, rel. humidity: 60-65%) and in accordance with international guidelines for animal welfare. Non-infected controls were housed separately from infected animals. Nutrition included commercial milk replacers and coarse meal. Water and hay were supplied *ad libitum*.

### Study design

#### Non-infected controls

Seven calves served as non-infected controls.

At the age of 3 months, BALF was sampled from all animals for flow cytometric analysis. Within the next four months, BALF was again sampled up to three times from each animal and BALF cells were stored for quantitative real time reverse transcription PCR (RT-PCR) of BALF cells at -80°C. The 17 BALF samples from non-infected controls originated from 7 animals, four animals were sampled three times, two animals were sampled twice and one animal was sampled once. For bronchoscopy, animals were sedated with xylazine (Rompun 2%, Bayer Vital GmbH, Leverkusen, Germany) and bronchoalveolar lavage was performed endoscopically in the standing animal, fluid used and further preparation have been described previously [16].

From two animals, lung tissue was sampled by transbronchial lung biopsy [19], and from another two animals, lung tissue was sampled at necropsy as described [16]. Tissue samples were stored at -80°C until RT-PCR analysis.

All animals remained clinically healthy during the time they were included in the study and the two animals that underwent necropsy showed no lesions or other pathological signs either.

#### Infected animals

Inoculation of 50 animals with 10^8^ ifu *C*. *psittaci*, strain DC15 was performed intrabronchially as described previously [14,19]. The challenge strain was isolated from an aborted calf fetus in 2002 and, therefore, assumed to be suitable for the investigation of chlamydial infections in bovines [14,20]. At time point of inoculation, animals were aged 6–8 weeks. Starting 36 h after inoculation and lasting until 13 dpi, 25 of these infected animals received either of the following daily antibiotic treatments: azithromycin (n = 7 animals), erythromycin (n = 6), azithromycin in combination with rifampicin (n = 6), and erythromycin in combination with rifampicin (n = 6). Dosages and application, as well as additional data on the clinical course, findings at necropsy, acute phase reaction, differential blood and BALF cell count and pathogen detection in this group of animals can be found in a recent publication [17]. Successful infection of all inoculated animals was reported previously [14,18] and consequently the designation “infected animals” is used throughout this manuscript to describe this group of animals. Application of Kruskal-Wallis test on all data obtained for the parameters described in this manuscript yielded no significant differences comparing the different treatment groups and the untreated group (*P* > 0.05). Data of treated and untreated animals were drawn together for further analysis to form a comprehensive data set comprising data from all infected animals. Samples and animal numbers at different time-points are given in Table 1. From 30 infected animals, venous blood was sampled into EDTA containers at seven time points (EDTA Primavette, 2.6 mL, KABE LABORTECHNIK GmbH, Nümbrecht-Elsenroth, Germany) and prepared for flow cytometric analysis immediately after sampling. From the same animals, BALF was endoscopically sampled at 4 and 9 dpi under general anesthesia as described elsewhere [19]. All animals were euthanized 14 dpi and BALF was sampled from the exenterated lung as described [16]. Also, one piece of macroscopically normal lung and one piece of inflamed lung tissue were sampled from each animal. BALF cells for flow cytometric analysis were prepared immediately, whereas BALF cells and lung tissue for RT-PCR were stored at -80°C until further processing. From the remaining 20 infected animals, 2.5 mL of venous blood was collected into PAXgene Blood RNA tubes (Becton Dickinson GmbH, Heidelberg, Germany) for RNA stabilization. Samples were incubated at room temperature for 4 hours and stored at –20°C until analysis.

**Table 1:** Time table illustrating the number of samples taken from calves inoculated with *C*. *psittaci* and methods used to assess various parameters.

subgroup of infected animals	sample	method	time											
			ai		hours (h)/days (d) post inoculation (pi)									
			-24 h	-1 h	4 h	1 dpi	2 dpi	3 dpi	4 dpi	5 dpi	7 dpi	9 dpi	10 dpi	14 dpi
30 animals (25 treated, 5 untreated)	blood	flow cytometry		n = 30		n = 30	n = 30	n = 30		n = 30	n = 30		n = 20	
	BALF	flow cytometry, RT-PCR							n = 30			n = 30		n = 30
	lung tissue	RT-PCR												n = 30
20 animals (untreated)	blood	RT-PCR	n = 20		n = 20	n = 18	n = 18	n = 13	n = 8					

a.i.: ante inoculation; BALF: bronchoalveolar lavage fluid; RT-PCR: real time reverse transcription PCR.

### Flow cytometry analysis of blood and BALF cells

**Fig 1:**
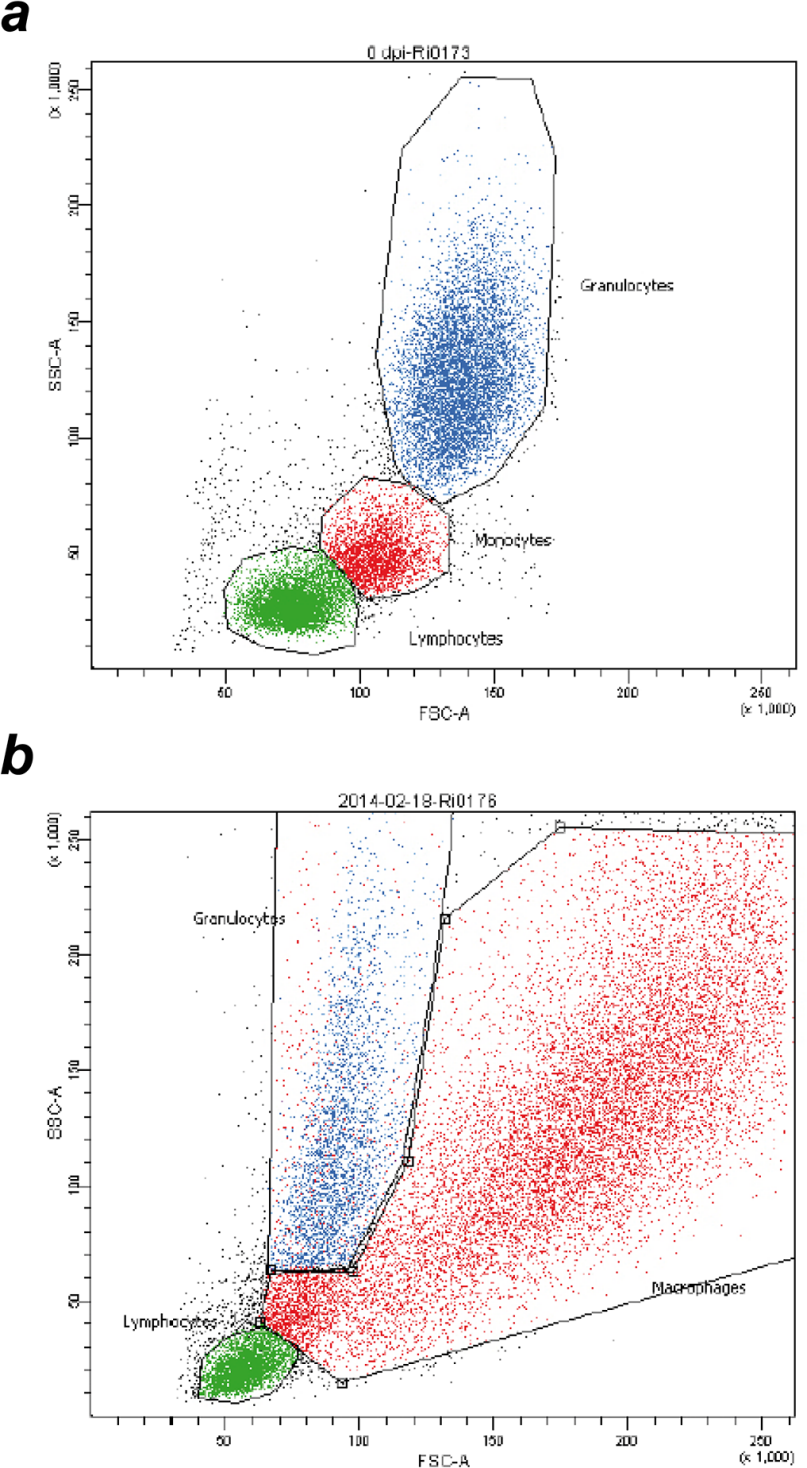
Gating of leukocyte subpopulations by flow cytometry. Forward versus sideward scatter plots of blood leukocytes (a) and bronchoalveolar fluid (BALF) cells (b).

**Fig 2:**
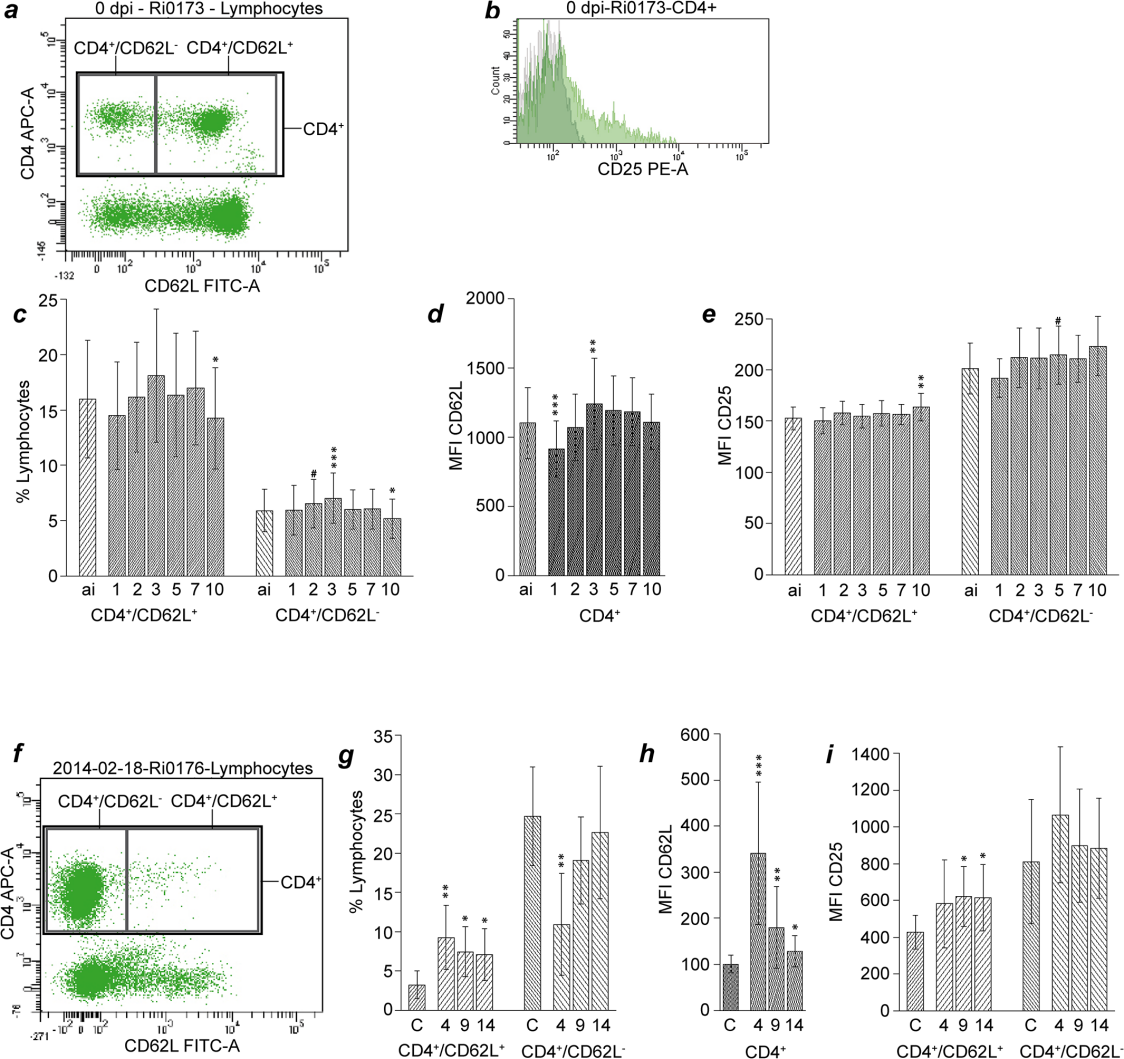
Analysis of CD4^+^ blood and BALF lymphocytes after *C*. *psittaci* inoculation of calves. For blood lymphocytes, definition of subpopulations (a), representative CD25 expression (b), proportions of subpopulations (c), CD62L expression on CD4^+^ cells (d), and CD25 expression on CD4^+^/CD62L^+^ and CD4^+^/CD62L^-^ cells (e) are given. All post-inoculation values were compared to ai-values with the Wilcoxon signed rank test, and then *P-*values were adjusted according to Holm (# 0.05 < *P* ≤ 0.1; * 0.01 < *P* ≤ 0.05; ** 0.001 < *P* ≤ 0.01; *** *P* ≤ 0.001). For BALF lymphocytes, definition of subpopulations (f), proportions of subpopulations (g), CD62L expression on CD4^+^ cells (h), and CD25 expression on CD4^+^/CD62L^+^ and CD4^+^/CD62L^-^ cells (i) are given. All values of infected animals were compared to values of healthy controls using the Mann-Whitney U test with Holm adjustment of *P*-values. Data are presented as mean and standard deviation obtained with samples from n = 30 animals (n = 20 at 10 dpi). ai: one hour before inoculation; C: healthy control animals (n = 7); numbers below x-axis refer to days post inoculation; MFI: mean fluorescence intensity.

**Fig 3:**
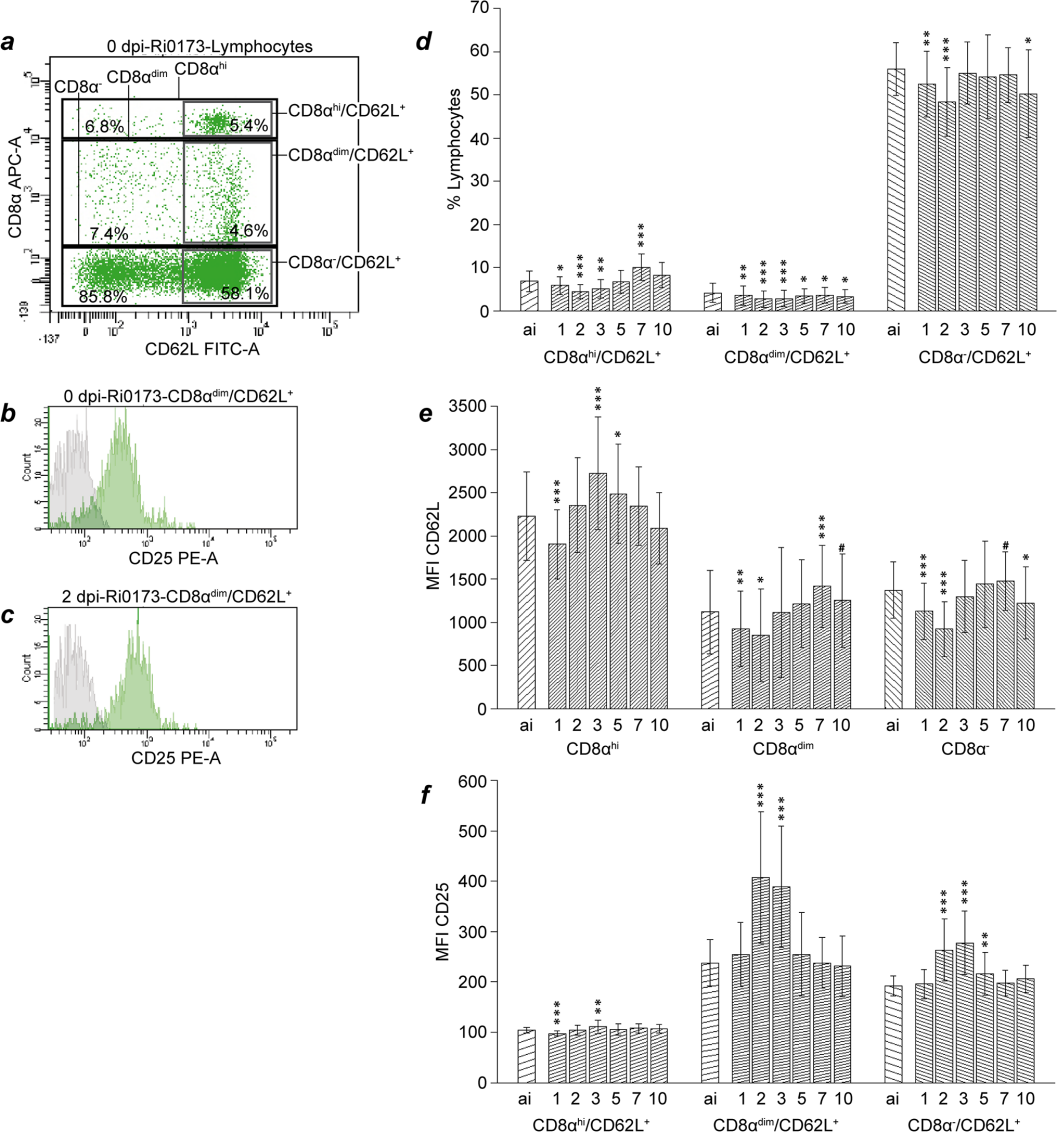
Analysis of CD8α^+^ blood lymphocytes after *C*. *psittaci* infection of calves. Definition of subpopulations (a), representative CD25 expression (b and c), proportions of subpopulations (d), CD62L expression (e) and CD25 expression (f) on subpopulations are given. For labelling of x-axis, sample numbers and statistical analysis see legend to Fig 2.

Thirty-five mL of BALF was centrifuged at 300 × *g* for 20 min. The supernatant was discarded and the cell pellet resuspended in 800 μL phosphate buffered saline solution (PBS). One-hundred μL of whole blood or resuspended BALF cells were incubated with primary antibodies (Table 2) and, if those were not directly labelled, secondary antibodies for 30 minutes at room temperature in the dark. Erythrocytes were lysed using BD FACS Lysing solution (Becton Dickinson GmbH, Heidelberg, Germany) according to the manufacturer’s instructions, samples were centrifuged at 300 × *g* for 5 minutes and supernatant was decanted. Cells were resuspended and fixed with 1 mL 4% formaldehyde solution in PBS for 30 minutes at room temperature, subsequently washed with 3 mL PBS and resuspended in 100 μL PBS for flow cytometry analysis with a FACS Canto II (Becton Dickinson GmbH, Heidelberg, Germany) with a blue (488 nm) and a red (635 nm) laser. Data analysis was performed with the BD FACSDiva software (Version 6.1.3, BD Biosciences). Doublets were excluded from the data using a FSC-height vs. FSC–width dot plot. Prior to the study, isotype controls were performed to confirm specificity of antibody binding. During the study, unstained cells served as negative controls for cells stained with directly labelled antibodies while cells incubated with secondary antibodies only served as negative controls for indirectly immunolabelled samples. Gates defining leukocyte subpopulations were set according to forward versus sideward scatter characteristics of the events (Fig 1). Unstained blood cells were measured with the same instrument settings as BALF cells and gates in the forward versus sideward scatter plot of BALF leukocytes were set similar to the ones defining blood leukocytes. The appropriateness of the BALF lymphocyte gate position was confirmed by back-gating showing that CD4^+^ events exclusively clustered in this gate. Prior to further analysis, events yielding a significant autofluorescence signal (FL-2 versus FL-4) indistinguishable from that of presumptive alveolar macrophages were excluded from the granulocyte gate by Boolean gating. Fluorescence gates were set according to the negative controls defining less than 2% of the cells as positive. Lymphocyte subpopulations were defined according to expression of CD4, CD8α and CD62L (Figs 2A and 2F and 3A). Representative examples of CD25 expression on CD4^+^ and CD8α^dim^ blood lymphocytes are given in Fig 2B and in Fig 3B and 3C, respectively. Absolute cell numbers were calculated based on differential blood counts and total leukocyte numbers presented in detail in a previous manuscript [17] and therefore not included herein. Intensity of surface marker expression is deduced from recording geometric means of fluorescence intensities (MFI).

**Table 2:** Antibody combinations and dilutions used for flow cytometry staining.

Tube	Specificity	Clone name and Literature	Isotype	Labelling	Dilution	cells stained
1	CD8α^a^	CC63 [21]	mIgG_2a_	AF647^c^	1:100	blood
	CD62L^a^	CC32 [22]	mIgG_1_	FITC^c^	1:100	
	CD25^a^	IL-A111 [23]	mIgG_1_	PE^c^	1:100	
2	CD4^a^	CC8 [21]	mIgG_2a_	AF647^c^	1:100	blood, BALF
	CD62L^a^	CC32 [22]	mIgG_1_	FITC^c^	1:100	
	CD25^a^	IL-A111 [23]	mIgG_1_	PE^c^	1:100	
3	CD4^b^	CACT138A [24]	mIgG_1_	a-IgG_1_-PE^d^	1:1000	blood
	MHCII^a^	49.1 [25]	mIgG_2a_	FITC^c^	1:500	
4	CD14^b^	CAM36A [26]	mIgG_1_	a-IgG_1_-PE^d^	1:500	blood
	MHCII^a^	49.1 [27]	mIgG_2a_	FITC^c^	1:500	
5	B-B11^b^ *	LCT30A[28]	mIgG_1_	a-IgG_1_-PE^d^	1:500	blood, BALF
6	CD11b^b^	MM12A [28]	mIgG_1_	a-IgG_1_-Alexa488^e^	1:500	blood

^a^from AbD Serotec, Kidlington, UK

^b^from VMRD, Pullman, WA, USA

^c^directly labelled

^d^from SouthernBiotech, Birmingham, AL, USA

^e^from Life Technologies, Carlsbad, CA, USA

*stains B Lymphocytes; final dilution of all secondary antibodies was 1:250.

### Quantification of gene transcripts by RT-PCR in blood

Total RNA was extracted from blood samples and purified using the PaxGene Blood RNA Kit (Qiagen, Hilden, Germany) according to the manufacturer’s recommendations. RNA concentration and purity were spectrophotometrically determined using a Nanodrop 1000 spectrophotometer (PEQLAB Biotechnologie GmbH, Erlangen, Germany) at 260 nm and 280 nm wavelength. The integrity and size distribution of total RNA was checked by denaturing formaldehyd agarose gel electrophoresis and ethidium bromide staining. Using the Reverse Transcriptase Core kit (Eurogentec, Koeln, Germany), 100 ng of total RNA were transcribed into cDNA with a final concentration of 5 mM MgCl_2_, 500 μM of each DNTP, 2.5 μM random nonamers, 0.4 U/μl RNAse inhibitor and 1.25 U/μl EuroScript reverse transcriptase in a reaction volume of 10 μl, respectively. The samples were initially incubated at 25°C for 10 min, followed by the transcription step at 48°C (30 min) and enzyme inactivation at 95°C (5 min). Primer sequences were designed to bind specifically to bovine cDNA using the Beacon Designer software (Premier Biosoft, Palo Alto CA, USA) according to published bovine cytokine mRNA sequences (NCBI). To prevent amplification of genomic DNA, primers (Eurogentec) were selected that anneal at intron splice junctions. Housekeeping genes included for normalization were *B2M*, *ACTB*, and *YWHAZ* out of a panel of nine genes (GeNorm; for primer sequences and full names of gene products see Table 3). Quantitative Real Time-PCR was performed as previously described with the CFX96 Real Time-PCR thermocycler (BioRad, München, Germany) using the qPCR Mastermix Plus for SYBR-Green I No Rox (Eurogentec) according to the manufacturer’s instructions [29]. The Δ*Ct* was calculated by subtracting the mean of the *Ct* values of the housekeeping genes from the *Ct* value of the target gene. Results are expressed as 40 – Δ*Ct* values.

**Table 3:** Real Time-PCR primer sequences and NCBI accession numbers.

mRNA target	Gene product	NCBI accession No.	Primer sequence [5’– 3’]	
*IL1B*	Interleukin 1β	NM_174093	forward	CCAGCTTCTGATGAGCAACCA
			reverse	CAGATGCGCCTGCTTCTAGG
*IL2*	Interleukin 2	M12791.1	forward	CCTCAACTCCTGCCACAATGTA
			reverse	AAATCCAGCAGCAATGACTTCA
*IL2RA*	Interleukin 2 receptor α	NM_174358	forward	CCTGCTGAAAGCACCTGCAT
			reverse	CTGCGTCATCTGAAGCCTGAC
*IL6*	Interleukin 6	NM_173923	forward	TCCCGCTTCACAAGCGCCTTC
			reverse	AGGGGACCCGGGGTAGGGAA
*IL10*	Interleukin 10	NM_174088	forward	TGACATCAAGGAGCACGTAA
			reverse	TCTCCACCGCCTTGCTCTT
*IL12B*	Interleukin 12B	NM_174356	forward	GTGGAGTGTCAGGAGGGCAGC
			reverse	GGTGGGTCTGGTTTGATGATGTCC
*IFNG*	Interferon γ	NM_174086	forward	CAAATTCCGGTGGATGATCTG
			reverse	TCTGACTTCTCGTCCGCTTTC
*TNF*	Tumor necrosis factor	NM_173966	forward	GCCCACGTTGTAGCCGACATCA
			reverse	CACCGTTGGCCATGAGGGCA
*TLR2*	Toll-like receptor 2	NM_174197	forward	TGAGATGGTTGGATGGCATCA
			reverse	AACCTCATGGACTGCAGCACA
*HSPA1A*	Heat shock 70kDa protein 1A	NM_174550	forward	CAAGAGGAAGCACAAGAAGGA
			reverse	GCTGGACGACAAGGTTCTCT
*B2M*	β-2-microglobulin	NM_173893	forward	GAGCCGCTCACTCCGCCAC
			reverse	TGGAGGACGCTGGATGGCGT
*ACTB*	Actin β	NM_173979	forward	AGCAGATGTGGATCAGCAAG
			reverse	CAGCTAACAGTCCGCCTAGAA
*YWHAZ*	Tyrosine 3-monooxygenase/tryptophan 5-monooxygenase activation protein ζ	NM_174814	forward	ACTCCGGACACAGAACATCCAGTCA
			reverse	CCCTCCAAGATGACCTACGGGCT
*GAPDH*	Glyceraldehyde-3-phosphate dehydrogenase	NM_001034034.2	forward	AATTTGGCTACAGCAACAGGG
			reverse	AACTCTTCCTCTCGTGCTCC
*CXCL8*	Interleukin 8	NM_173925.2	forward	AAAGTGGGTGCAGAAGGTTG
			reverse	CCACACAGAACATGAGGCAC
*TNFRSF9*	Tumor necrosis factor receptor superfamily, member 9	NM_001035336.2	forward	GTGGCTACTGTGCTATTGGT
			reverse	GTCCACTTGTGCTGGAGAAA

### Quantification of gene transcripts by RT-PCR in BALF cells and lung tissue

For analysis of transcription of *CXCL8* and *TNFRSF9* (for full names of gene products see Table 3), BALF cells were pelletized for 10 min at 400 × g/ 4°C and RNA was extracted using the peqGOLD Total RNA Kit including an on-membrane DNase I digestion (PEQLAB Biotechnologie GmbH, Erlangen Germany). Tissue was cut into pieces, lysed chemically and mechanically (Tissue Lyser LT, Qiagen) and processed as BALF cells. An additional DNAse I digestion was done by using the peqGOLD DNase I Kit (PEQLAB Biotechnologie GmbH, Erlangen, Germany) and Recombinant RNasin Ribonuclease Inhibitor (Promega GmbH, Mannheim, Germany). RNA was precipitated according to the manufacturer’s protocol and dissolved in 50 μl of nuclease-free water. The RNA-concentration was determined photospectrometrically (NanoDrop, PEQLAB) and 1000 ng RNA were applied to the reverse transcription reaction (Reverse Transcription System, Promega Corporation, Madison, USA). Incubation took 25 min at 42°C followed by 5 min inactivation at 99°C. cDNAs were diluted 1:10 in DEPC-water (Carl Roth GmbH + Co. KG, Karlsruhe, Germany) and stored at -20°C. Real time RT-PCRs were run on the SmartCycler II (Cepheid, Maurens-Scopont, France) with a three step profile: initial denaturation 95°C, 120 sec, denaturation 95°C, 10–20 sec, annealing, 59°C (tissue)/ 63°C (BALF), 20 sec, elongation, 72°C, 10–20 sec, 45 cycles, melting curve (60 to 95°C). PCR reaction mixes were composed as follows: 10 μl 2 x KAPA SYBR FAST QPCR MasterMix Universal (KAPABIOSYSTEMS, Boston, USA), 0.4 μl of each primer fwd and rev (10μM), 4.2 μl PCR- water, 5 μl diluted cDNA. Primer sequences are given in Table 3. Calculation of the target gene mRNA level in relation to *GAPDH* (relative abundance) was done by using the following formula:

$$2^{-(Ct\left(target\right)-Ct(GAPDH))}\;.$$

### Statistical methods

R [30] has been used for statistical evaluation. Data were tested for normal distribution (Shapiro-Wilk-Test) and not all data were normally distributed, sometimes data from different days had different distributions. Therefore non-parametrical tests were chosen for statistical evaluation of all data. The Wilcoxon signed rank test with zero handling according to Pratt from the package coin [31] and Holm adjustment was used for comparing pre- with post-inoculational values. For comparison of infected animals with non-infected controls, the two-sided Mann-Whitney U test with Holm adjustment was used. Values of *P* ≤ 0.05 were considered significant. Values of 0.05 ≤ *P* < 0.1 were regarded as tendencies and are given in the graphs. Unless stated differently, data are given as mean and standard deviation (SD). In ‘Box and Whiskers plots’, outlier values (circles) are more than 1.5 times of the length of a box away from the median. In bar plots, the mean is represented by the length of the bar; the error bar represents the standard deviation.

## Results

### Kinetics of leukocyte subpopulations and activation marker expression in peripheral blood

Flow cytometric analysis of infected and non-infected calves revealed significant changes in blood cell composition and especially in activation marker expression by T cell subsets, monocytes and granulocytes depending on the infection status and over time.

Prior to inoculation, 21.9 (mean SD 6.3) % of all blood lymphocytes were CD4^+^. With the onset of clinical signs at 3 dpi, this number significantly increased to a maximum of 25.1 (6.8) %, before it returned to ante-infection (ai) level at 7 dpi and even significantly dropped below that on 10 dpi (19.4 (5.7) %). Of CD4^+^ cells, a major portion of 72.2 (6.5) % co-expressed CD62L, which remained stable after inoculation with *C*. *psittaci*. The proportion of CD4^+^/CD62L^+^ cells within the lymphocyte gate also remained stable, despite a significant drop below ai values on 10 dpi (Fig 2C). The proportion of CD4^+^/CD62L^-^ lymphocytes followed a similar trend over time, although values were slightly but significantly elevated at 3 dpi. Variations in total lymphocyte counts [17] led to a significant decrease of the total numbers of these two populations at 2 and 10 dpi (data not shown). As deduced from mean fluorescence intensities, average CD62L expression per single CD4^+^ cell significantly decreased below ai values at 1 dpi to rise again to its maximum at 3 dpi, where it significantly exceeded ai values (Fig 2D). From 5 to 10 dpi, CD62L expression was comparable with baseline level. Of note, CD4^+^/CD62L^-^ cells expressed remarkably more CD25 on a per cell basis than CD4^+^/CD62L^+^ blood lymphocytes before and after infection with *C*. *psittaci* (Fig 2E). CD25 expression remained virtually unchanged in both lymphocyte subsets, the only statistically significant differences to ai values being an increase at 10 dpi on CD4^+^/CD62L^+^ lymphocytes. Only a minor subset (2.1 (1.2) %) of CD4^+^ blood lymphocytes was MHC-II^+^ before inoculation, but this subset doubled to a maximum of 4.3 (2.2) % on 2 dpi and remained significantly elevated over baseline values until 5 dpi (S1 Fig). Similarly, total numbers of this population increased from 2.7 (1.9) × 10^4^ cells × mL^-1^ before inoculation to a maximum of 4.4 (3.0) × 10^4^ cells×mL^-1^ at 3 dpi, which was significantly higher than baseline values from 2 to 5 dpi.

Before inoculation, 7.9 (2.5) % of lymphocytes were CD8α^hi^ and 9.1 (3.3) % were CD8α^dim^. As early as 2 dpi, only 5.1 (1.8) % and 6.8 (2.6) % of lymphocytes belonged to the CD8α^hi^ and CD8α^dim^ subset, respectively; these values being significantly lower than baseline. The proportion of CD8α^dim^ cells remained significantly below baseline level until 10 dpi, whereas the proportion of CD8α^hi^ lymphocytes significantly rose to a maximum (11.8 (3.3) %) on 7 dpi. Total numbers (data not shown) and proportions of CD8α^hi^/CD62L^+^, CD8α^dim^/CD62L^+^, and CD8α^-^/CD62L^+^ lymphocytes dropped significantly below baseline levels after inoculation, with only the CD8α^hi^/CD62L^+^ population showing a short but significant increase on 7 dpi (Fig 3D). Regardless of chlamydial infection, expression of CD62L was almost twice as high on CD8α^hi^ as on CD8α^dim^ and CD8α^-^ cells (Fig 3E). After an initial drop below baseline level on 1 dpi, expression of CD62L on the CD8α^hi^ population increased significantly to a maximum on 3 dpi to then drop again, reaching ai values at 7 dpi. With some delay, expression of CD62L on CD8α^dim^ and CD8α^-^ cells declined significantly below baseline level after inoculation, being minimal at 2 dpi before it rose to baseline levels again. While CD8α^hi^ lymphocytes in general were characterized by high numbers of CD62L molecules on their surface, at least the CD8α^hi^/CD62L^+^ subset expressed significantly less CD25 than CD8α^dim^/CD62L^+^ and CD8α^-^/CD62L^+^ cells (Fig 3F). In some contrast to CD8α^hi^/CD62L^+^ cells, CD25 expression by CD8α^dim^/CD62L^+^ cells and, to a lesser extent, by CD8α^-^/CD62L^+^ lymphocytes significantly peaked 2 and 3 dpi (Fig 3B,3C and 3F).

Absolute numbers of B-B11^+^ blood lymphocytes stayed stable (ai value: 1.9 × 10^6^ (6.5 × 10^5^) cells × mL^-1^) throughout the entire study, but their proportion within total blood lymphocytes was significantly increased from 1 to 3 dpi with a maximum of 46.4 (9.5) % on 2 dpi compared to 31.8 (8.7) % before inoculation.

**Fig 4:**
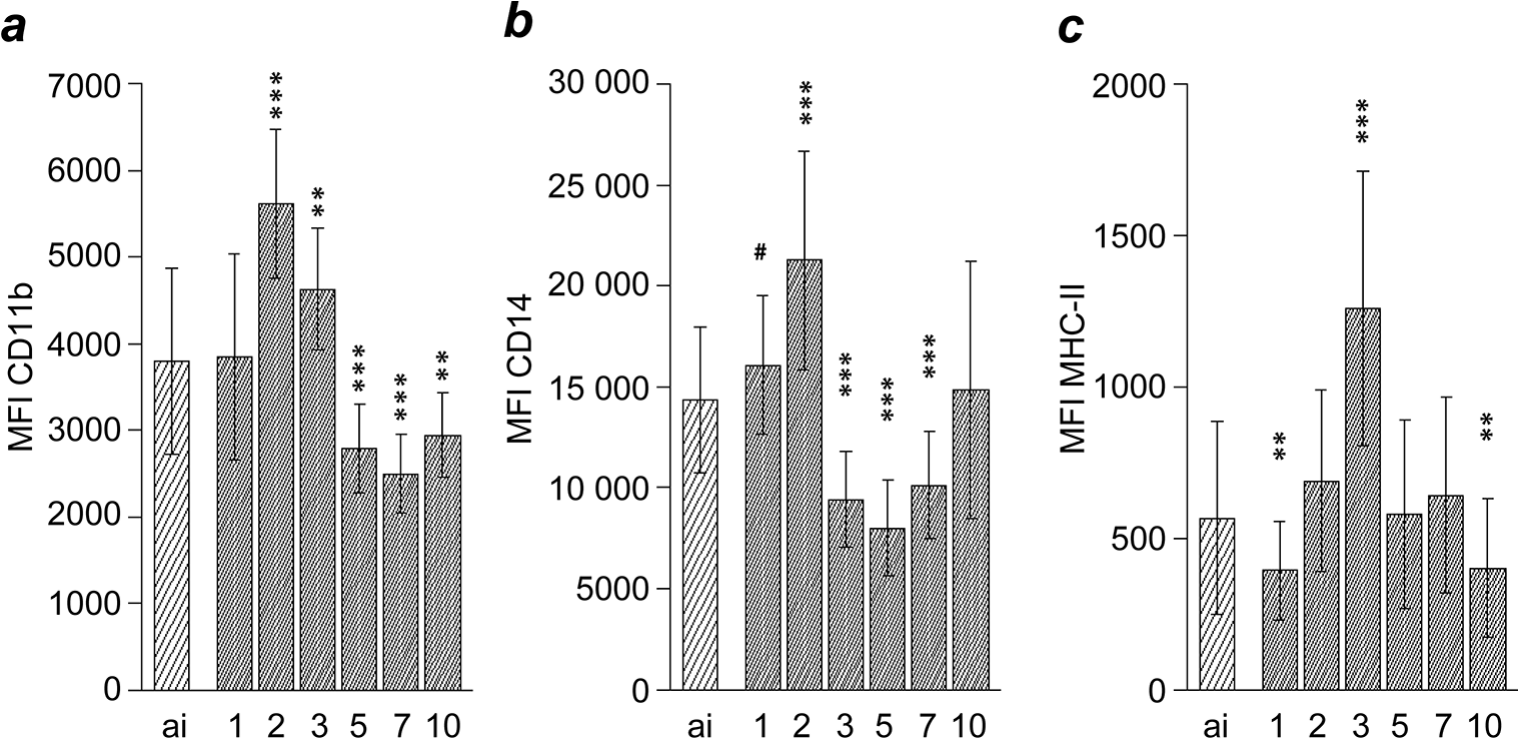
Analysis of blood monocytes after *Chlamydia psittaci* inoculation of calves. Expression intensity of CD11b (a) is given for all cells in the monocyte population, whereas expressions intensities of CD14 (b) and MHC-II (c) are given only for the CD14^+^ cells in the monocyte population. For labelling of x-axis, sample numbers and statistical analysis see legend to Fig 2.

After inoculation, CD11b expression by blood monocytes significantly increased to a maximum on 2 dpi to decline significantly below ai values until 5 dpi, where it remained until the end of the study (Fig 4). Similarly, a significant increase of CD14 expression on CD14^+^ blood monocytes until 2 dpi was followed by a drop to values significantly below baseline levels from 3 to 7 dpi. Expression of MHC-II on CD14^+^ monocytes followed the opposite kinetics in that it increased threefold at 3 dpi after a transient decrease and before dropping below ai values again.

**Fig 5:**
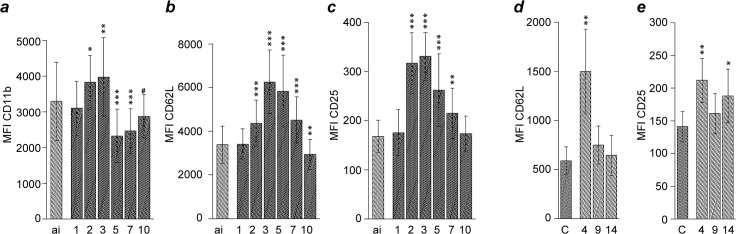
Analysis of blood and BALF granulocytes after *Chlamydia*. *psittaci* inoculation of calves. On blood granulocytes (a, b, c) and BALF (d, e) granulocytes, the expression intensity of CD11b, CD62L, and CD25 is shown. For labelling of x-axis, sample numbers and statistical analysis see legend to Fig 2.

With the onset of clinical signs, expression of CD11b on blood granulocytes was characterized by a rise to a significant maximum on 3 dpi, followed by a sharp drop significantly below ai values on 5 dpi (Fig 5). Expression of CD62L on blood granulocytes significantly increased to a maximum on 3 dpi to then drop below ai values on 10 dpi. CD25 expression on blood granulocytes was significantly higher than baseline values from 2 to 7 dpi, with the maximal value on 3 dpi being almost twice as high as before inoculation.

### Kinetics of lymphocyte subpopulations and activation maker expression in BALF

In healthy controls, 27.5 (7.0) % of all BALF lymphocytes were CD4^+^. On 4 dpi, this portion was significantly lower (20.1 (8.1) %) in infected animals and returned to values of healthy controls by 9 dpi. In the BALF of infected animals, a significantly higher percentage of lymphocytes was CD4^+^/CD62L^+^ than in healthy controls, with the maximum at 4 dpi (Fig 2G). At the same time, the percentage of CD4^+^/CD62L^-^ lymphocytes in infected animals was significantly below values of healthy controls, but almost reached this level by 14 dpi. Expression of CD62L on CD4^+^ BALF lymphocytes was three times higher in infected animals 4 dpi than in healthy controls and decreased until 14 dpi, where it was still significantly elevated over values in healthy controls (Fig 2H). However, CD62L expression by CD4^+^ BALF lymphocytes was considerably lower than that of CD4^+^ blood lymphocytes. In contrast, CD4^+^ BALF lymphocytes, with CD4^+^/CD62L^-^ cells in particular, expressed considerably higher numbers of CD25 molecules than CD4^+^ blood lymphocytes (Fig 2I). CD4^+^/CD62L^+^ BALF lymphocytes, expressing slightly less CD25 than CD4^+^/CD62L^-^, presented with increased CD25 expression in infected animals at 9 and 14 dpi compared to healthy controls, whereas CD4^+^/CD62L^-^ cells exhibited a distinct, but not statistically significant increase of CD25 expression at 4 dpi as compared to 9 and 14 dpi and healthy controls.

Proportions of CD8α expressing cells in the BALF did not change during the study and did not differ between infected animals and healthy controls and infection did not influence the proportion of B-B11^+^ BALF lymphocytes (data not shown).

BALF granulocytes of infected animals showed an almost threefold increase in CD62L expression at 4 dpi (Fig 5D). Nevertheless, baseline values of CD62L expression on blood granulocytes was six times as high as on BALF granulocytes. In infected animals, BALF granulocytes expressed higher numbers of CD25 molecules than respective cells from healthy controls, differences were statistically significant at 4 and 14 dpi (Fig 5E).

### Expression of immune mediators and their receptors in blood, BALF and lung tissue

**Fig 6:**
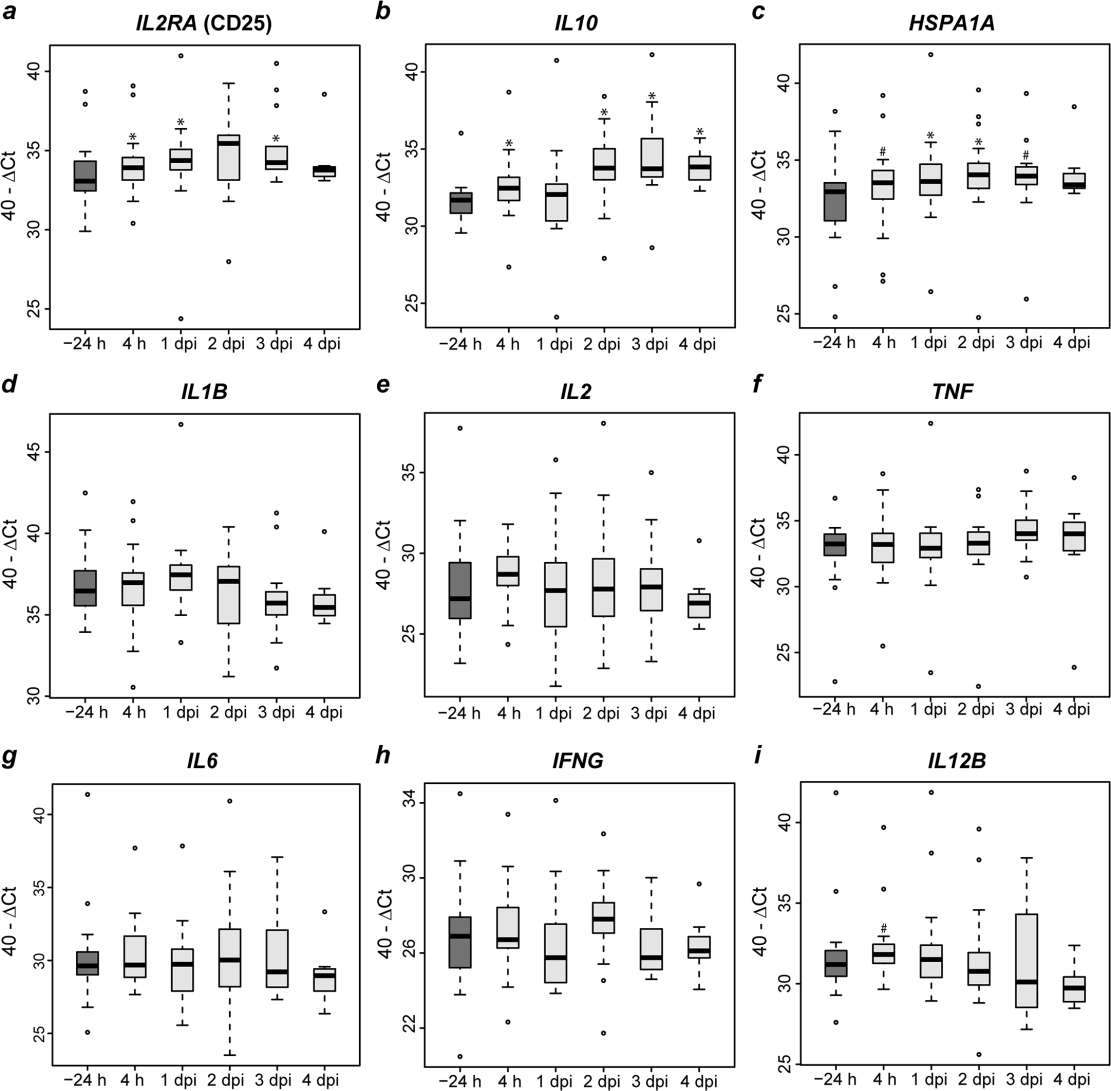
Quantitation of selected cytokine and receptor specific gene transcripts in blood after *Chlamydia psittaci* inoculation of calves. Amounts of mRNA are presented box and whisker plots of 40-ΔCt values. -24 h: 24 hours before inoculation (n = 20); 4 h: 4 hours after inoculation (n = 20); 1 dpi and 2 dpi: n = 18; 3 dpi: n = 13; 4 dpi: n = 8. Post-inoculation values of animals were compared to pre-inoculation values of the same animals with the Wilcoxon signed rank test, then *P-*values were adjusted according to Holm (# 0.05 < *P* ≤ 0.1; * 0.01 < *P* ≤ 0.05).

Amounts of mRNA for *IL2RA* (encoding for CD25), *IL10* and *HSPA1A* significantly increased in blood as early as 4 h after inoculation compared to pre-inoculation values (Fig 6A–6C) whereas amounts of *IL1B*, *IL2* and *TNF* specific mRNA only moderately increased or remained unaltered as for *IL6* (Fig 6D–6G) and *TLR2* (data not shown). *IFNG* specific mRNA quantitatively varied over time (Fig 6H). In contrast, amounts of RNA for *IL12B* dropped below pre-inoculation values after a transient increase (Fig 6I).

**Fig 7:**
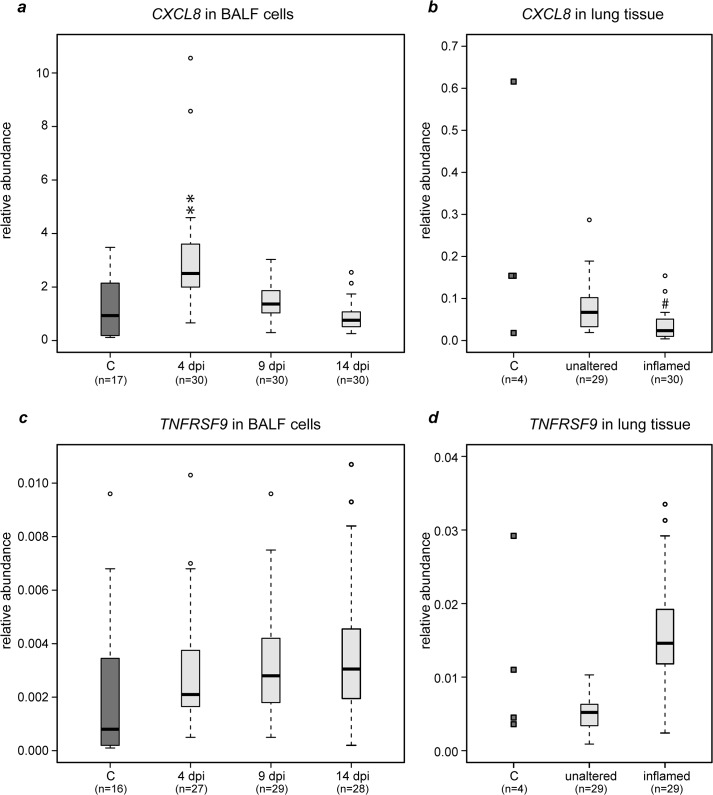
Quantitation of *CXCL8* and *TNFRSF9* specific mRNA in BALF cells and lung tissue after *Chlamydia psittaci* inoculation of calves. Levels of mRNA encoding for Interleukin 8 and TNFRSF9 in BALF cells, in macroscopically normal and in inflamed lung tissue sampled 14 days after inoculation (dpi) are given as relative abundance. C: healthy controls. All values of infected animals were compared to values of healthy controls using the Mann-Whitney U test with Holm adjustment of *P*-values (# 0.05 < *P* ≤ 0.1; ** 0.001 < *P* ≤ 0.01).

Compared to non-infected animals, the relative abundance of *CXCL8* mRNA (encoding for Interleukin 8) in BALF increased significantly by 4 dpi and returned to levels of non-infected controls by 14 dpi (Fig 7A). Analysis of tissue samples (14 dpi) revealed a lower expression of *CXCL8* in inflamed tissue compared to macroscopically unaltered tissue of both infected calves and non-infected controls (Fig 7B). BALF cells of infected animals exhibited slightly more *TNFRSF9* transcripts than BALF cells of non-infected calves (Fig 7C). Also, the relative abundance of this mRNA was higher in inflamed lung tissue than in macroscopically unaltered tissue and tissue of non-infected animals (Fig 7D).

## Discussion

To further our understanding of host response effector mechanisms, the bovine *C*. *psittaci* infection model was chosen to dissect the dynamics and effects of local and systemic immune responses. The finding that all animals regain clinical health within two weeks after experimental *C*. *psittaci* infection stresses the importance of anti-bacterial defense mechanisms inherent to the host to protect cattle from the detrimental effects of chlamydial infections [14,17]. Data Analysis allowed a graduation of the immune response to respiratory *C*. *psittaci* infection in different phases as (i) initial phase 4 h-1 dpi, (ii) peak phase of 2–3 dpi, (iii) recruitment phase 3–5 dpi, (iv) local phase 4–14 dpi and (v) normalization (7–10 dpi).

The *initial phase (4 h – 1 dpi)* was mainly characterized by an increase of transcripts of *HSPA1A*, *IL2RA* (syn. CD25), and *IL10* in peripheral blood. Even though calves developed first clinical signs at 1 dpi, which were paralleled by increases in total leukocyte numbers with neutrophilic granulocytes in particular [14–16], activation marker expression levels on the immune cells remained mainly unchanged or even decreased at this stage.

In the *peak phase of disease (2–3 dpi)*, when clinical signs were most severe and neutrophil counts in the blood where highest, expression of CD62L, CD25 and CD11b on blood granulocytes were significantly elevated. CD62L and CD11b are synthesized by bovine neutrophils during maturation in the bone marrow [32]. Furthermore, CD11b is stored in intracellular granules of neutrophils and is rapidly expressed on the cell surface upon activation [33]. High levels of CD62L and CD11b thus mirror the observed regenerative left shift of the granulocyte population in the blood of *C*. *psittaci*-infected calves [15,18] and indicate an activated state of these cells. CD25, the α-chain of the IL-2 receptor, was described very recently as novel inflammatory marker on granulocytes in blood and milk of dairy cows [34]. Although levels of CD25 expression on granulocytes were rather low as compared to lymphocytes in our infection model, we found its increase to be a reliable marker in all animals inoculated with *C*. *psittaci*.

Peripheral blood monocytes increased expression of CD11b and of CD14 and MHC-II on the CD14^+^ subset. Porcine monocytes up-regulate MHC-II *in vitro* in response to LPS [35], implying activation of blood monocytes after *C*. *psittaci* infection in our model. As migration of monocytes into the alveolar space is CD11b-dependent [36] and absolute numbers of alveolar macrophages are known to increase upon intrabronchial inoculation of *C*. *psittaci* [14], elevated CD11b expression on monocytes may have facilitated transmigration of activated monocytes into the lung. In bovines, only classical and intermediate monocytes express CD14, i.e. two populations with the highest capacity to phagocytize and to generate reactive oxygen species [27]. In *C*. *psittaci*-infected calves, LBP concentration in peripheral blood increases significantly [15–17]. LBP binds bacterial LPS and promotes its recognition by the CD14 receptor [37–39] which, together with β2-integrin (CD11b/CD18), forms the LPS-activation cluster on monocytes [40,41]. Pathogenesis of acute *Chlamydia*-induced respiratory disease in calves relies on bacterial replication in lung tissue as intrabronchial challenge of calves with heat-inactivated *C*. *psittaci* suspensions failed to cause sizable clinical and ultrastructural effects [14]. In the present study, elevated CD14 and CD11b expression by blood monocytes along with elevated LBP levels suggests that the early local and systemic events induced by viable chlamydiae have enabled the calves to pass through a phase of enhanced sensitivity to LPS, implicated in the pathogenesis of clinical sequelae.

As early as 2–3 dpi, first signs of T cell activation became detectable. The CD8α^dim^ and the CD8α^-^ populations decreased their CD62L expression, but the remaining CD62L^+^ cells in both subpopulations markedly increased expression of CD25. Both effects were reported to correlate with bovine lymphocyte activation *in vitro* [42,43]. In contrast to this, the CD4^+^ and the CD8α^hi^ population in the blood significantly increased the expression of CD62L after an initial drop, but did not change their CD25 expression on CD62L^+^ cells. We hypothesize that γδT cells are the subpopulation entering an activated state defined by an increase of CD25 and a decrease of CD62L expression. Indeed, the kinetics of CD62L and CD25 expression on CD8α^-^/CD4^-^/CD62L^hi^ cells strongly resembled that of CD8α^dim^/CD62L^hi^ cells, which almost exclusively consist of γδT cells [44], a prominent T cell population in bovines [45]. The activation of this population is MHC independent, allowing fast reactions to pathogens. γδT cells produce anti-inflammatory IL-10 *in vitro* [46] and evidence has been accumulating that these cells, rather than CD4^+^/CD25^+^/Foxp3^+^ lymphocytes, primarily function as regulatory T cells in ruminants [47]. In line with this, elevated levels of IL-10 mRNA in the blood of *C*. *psittaci*-infected animals from 4 h until 4 dpi coincided with the appearance of phenotypically activated γδT cells and the relatively short duration of acute disease and inflammatory signs.

Antigen-specific, MHC-dependent T cells disappeared from circulating blood shortly after infection when they become trapped by antigen-presenting cells within lymph nodes. Numbers of a small subset of CD4^+^ cells coexpressing MHC-II^+^, a marker of T cell activation in bovines *in vitro* [48], increased after inoculation, being maximal in *C*. *psittaci-*infected calves as early as 2 dpi. *HSPA1A* transcription peaked in blood during this phase of reaction. Deposition of viable *C*. *psittaci* in the bovine lung induced a systemic acute phase response and local inflammation, inducing activation of the γδT cell pool and possibly of a minor subpopulation of CD4^+^ cells.

The subsequent *recruitment phase* partially overlapped with the peak phase and was dominated by neutrophilic granulocytes in blood and BALF of calves challenged with *C*. *psittaci*. All animals developed an increase in total numbers and percentages of neutrophilic granulocytes in the blood with a regenerative left shift, i.e. an increase of banded forms, and an increase of numbers and percentages of both, banded and segmented forms in the BALF [14–17]. IL-8 represents a potent proinflammatory chemokine recruiting and activating neutrophilic granulocytes [49,50]. In the early stage of the disease (4 dpi), elevated amounts of *CXCL8* transcripts could be detected in BALF cells of infected animals, thus corroborating the hypothesis that IL-8 is produced in *C*. *psittaci*-infected tissue to recruit and activate neutrophils [51], as in other chlamydial infections [52–54]. The *CXCL8* mRNA levels of BALF cells were highest 4 dpi, when clinical signs were most obvious and most neutrophils could be detected in BALF. Later on, when animals restored clinical health and BALF cell counts returned to physiological values [16], *CXCL8* mRNA levels were comparable with non-infected controls. mRNA levels of *CXCL8* in inflamed and in macroscopically unaltered lung tissue were strikingly lower on a per cell basis compared to the level in BALF cells. This may be due to the late time point after infection and corresponds to the time-dependent decrease in BALF cells. Low *CXCL8* levels in inflamed tissue, as compared to healthy tissue of infected animals and tissue of non-infected controls, may be due to the fact that the inflamed tissue was partially necrotic and cellular autolysis affected the amounts of mRNA species under study [16,17].

BALF granulocytes showed increased expression of CD62L and CD25 on 4 dpi as compared to healthy controls. Granulocytes shed parts of the CD62L on their surface during the process of binding to and migrating through the capillary wall, likely explaining the lower CD62L expression observed for BALF as compared to blood cells of *C*. *psittaci*-infected calves. The decrease of CD62L expression over time in blood and BALF can be considered a result of increased CD62L shedding along with granulocyte transmigration and aging [36,55,56].

The phenotype of T cells found in the BALF of infected and non-infected calves in the *local phase (4–14 dpi)* was different from the one present in blood. Physiologically, there are more activated phenotypes in BALF than in blood lymphocyte populations of healthy cattle [57,58]. In our study, the CD4^+^/CD62L^-^ population makes up a higher percentage of total lymphocytes and is characterized by far higher CD25 expression than the corresponding population in the blood and can be regarded as an activated phenotype. The initial drop of the percentage of BALF lymphocytes of this population with an increase of CD25 expression at the same time could be the combined effect of premature apoptosis of local T cells and a selective attraction of activated T cells to the infection site. The decrease of CD4^+^ BALF lymphocyte percentage found here is in accordance with previous findings in a smaller group of *C*. *psittaci*-infected calves, where, compared to non-infected controls, significantly lower numbers of CD4^+^ lymphocytes per mm^2^ were detected by immunohistochemistry from 2 to 10 dpi [59]. The drop of activated T lymphocytes in the BALF could also be caused by the chlamydial infection itself since *in vitro* experiments showed that infection of human monocyte-derived macrophages with *C*. *trachomatis* induces apoptosis of co-cultured of T cells [60].

In the *normalization phase (7–14 dpi)*, animals regained clinical health, and surface marker expression on blood and BALF leukocytes returned to pre-inoculation values, implying that the transient immune response was sufficient to control the pathogen. It is accepted that the cellular rather than humoral response to chlamydial infections is responsible for host immunity [54]. The serological response to chlamydial infections often poorly correlates with pathogen detection, and studies on naturally and experimentally infected calves revealed that only two thirds of the animals are or become seropositive [9,14,18]. The present bovine model, when observation periods are extended beyond fourteen days, even offers the opportunity to further investigate antigen-specific T cell responses involved in the host defense against chlamydial infections.

## Conclusion

The present study addressed the immunological reaction of calves to intrabronchial inoculation with the intracellular pathogen *C*. *psittaci* over two weeks. Changes in leukocyte surface marker expression and cytokine transcription paralleled the clinical course of the disease induced by *C*. *psittaci*. However, an initial temporary increase of transcripts for selected immune mediators was followed by rapid activation of immune cells, with blood monocytes, granulocytes and T cell subsets each following distinct kinetics. This study provides a deep insight in local and systemic host response evoked by calves during the acute phase of respiratory *C*. *psittaci* infection. Especially the more comprehensive analysis of the activation status of systemic und local immune cells contributes to extending our knowledge on the immune defense in *Chlamydia*-infected calves. Future studies to unravel the possible relevance of the enigmatic CD25 antigen expression on bovine granulocytes after *Chlamydia* infection will be of great interest.

## Supporting Information

S1 Fig Expression of MHC-II on CD4^+^ blood lymphocytes after *C*. *psittaci* inoculation of calves. The percentage of MHC-II^+^ cells on CD4^+^ blood lymphocytes (a) and the number of MHC-II^+^/CD4^+^ cells per mL blood (b) is given. All post-inoculation values were compared to ai-values with the Wilcoxon signed rank test, and then *P-*values were adjusted according to Holm (# 0.05 < *P* ≤ 0.1; * 0.01 < *P* ≤ 0.05; ** 0.001 < *P* ≤ 0.01; *** *P* ≤ 0.001). Data are presented as mean and standard deviation obtained with samples from n = 30 animals (n = 20 at 10 dpi). ai: one hour before inoculation; numbers below x-axis refer to days post inoculation.
(TIF)

S1 File The ARRIVE Guidelines Checklist. (PDF)
