# Supplementary figures and images for: Kinetics of Local and Systemic Leucocyte and Cytokine Reaction of Calves to Intrabronchial Infection with Chlamydia psittaci
Source: PLoS One. 2015 Aug 7;10(8):e0135161. doi: 10.1371/journal.pone.0135161 (PMC4529195; doi:10.1371/journal.pone.0135161)

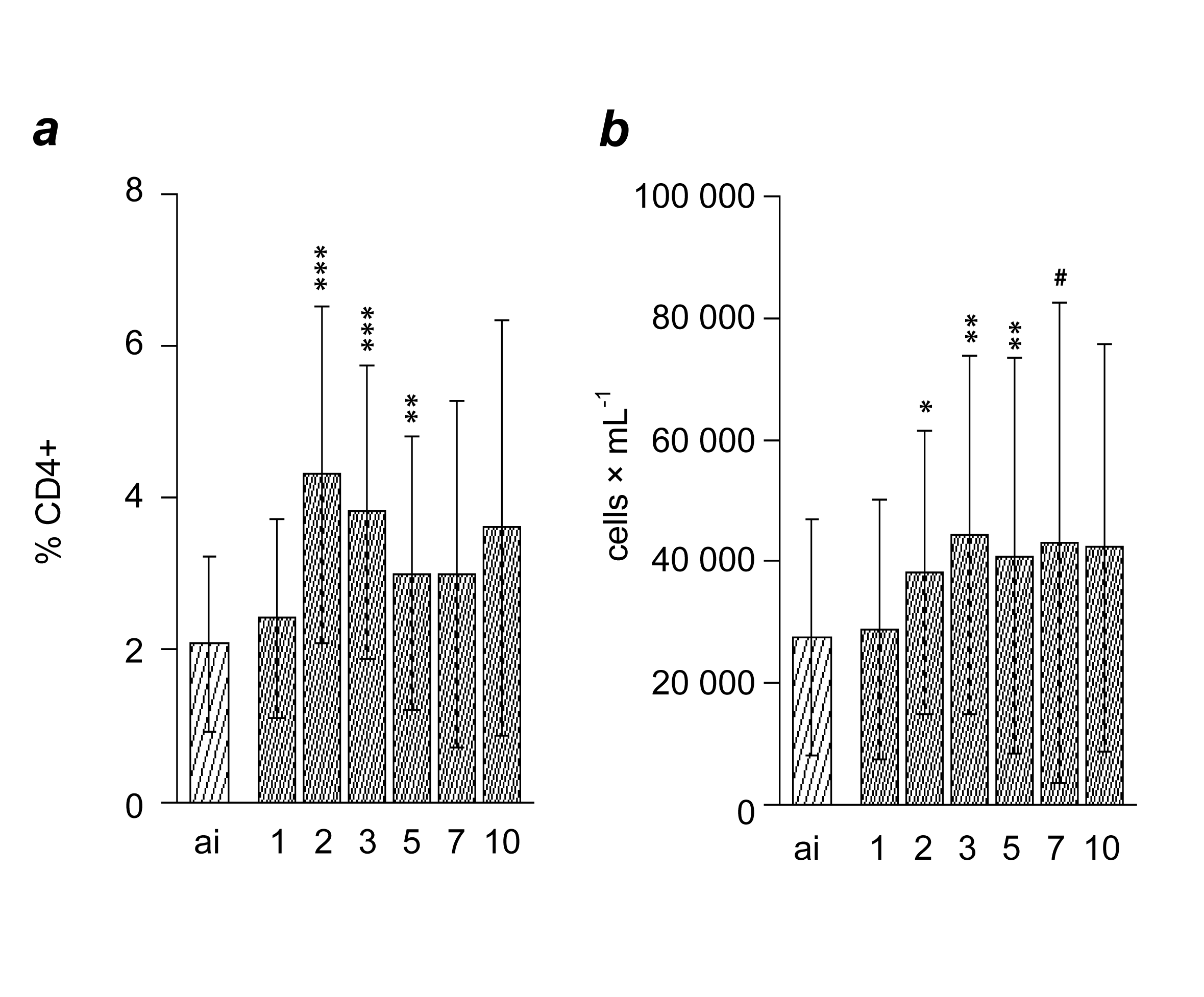

Supplement: S1 Fig — The percentage of MHC-II+ cells on CD4+ blood lymphocytes (a) and the number of MHC-II+/CD4+ cells per mL blood (b) is given. All post-inoculation values were compared to ai-values with the Wilcoxon signed rank test, and then P-values were adjusted according to Holm (# 0.05 < P ≤ 0.1; * 0.01 < P ≤ 0.05; ** 0.001 < P ≤ 0.01; *** P ≤ 0.001). Data are presented as mean and standard deviation obtained with samples from n = 30 animals (n = 20 at 10 dpi). ai: one hour before inoculation; numbers below x-axis refer to days post inoculation. (TIF) [file pone.0135161.s001.tif]
